# Positive correlation between snoring and dyslipidemia in adults: results from NHANES

**DOI:** 10.1186/s12944-023-01839-7

**Published:** 2023-06-16

**Authors:** Ying Tian, Dongna Li, Huijuan Mu, Sining Wei, Dong Guo

**Affiliations:** 1 Clinical Research Center, Affiliated Hospital of Shandong, University of Traditional Chinese Medicine, Jinan, China; 2 Department of Cardiology, Affiliated Hospital of Shandong University of Traditional Chinese Medicine, Jinan, China; 3 Drug clinical trial facility, Affiliated Hospital of Shandong University of Traditional Chinese Medicine, Jinan, China; 4 Prevention and treatment center, Affiliated Hospital of Shandong University of Traditional Chinese Medicine, Jinan, China; 5 Basic Medical School, College of Traditional Chinese Medicine, Shandong University of Traditional Chinese Medicine, Jinan, China

**Keywords:** Snoring, Dyslipidemia, Cross-sectional study

## Abstract

**Background:**

A few studies have shown that snoring, in certain populations, is associated with dyslipidemia. However, there are currently no large-scale national studies available that explore this association. Thus, for further clarification, studies using a large sample of the general population need to be conducted. This study aimed to explore this association using the National Health and Nutrition Examination Survey (NHANES) database.

**Methods:**

A cross-sectional survey was conducted using data from the NHANES database; 2005 to 2008 and 2015 to 2018 datasets were used (weighted to be representative of United States adults aged ≥ 20 years). Information on snoring status, lipid levels, and confounding factors were included. Logistic regression of the generalized linear model was used to analyze the relationship between snoring and dyslipidemia, and hierarchical analysis, interaction analysis, and sensitivity analysis were used to explore the stability of the results.

**Results:**

Data from 28,687 participants were analyzed, and 67% of the participants had some degree of snoring. The fully adjusted multivariate logistic regression results demonstrated that snoring frequency was significantly positively associated with dyslipidemia (*P* < 0.001 for linear trend). Adjusted odds ratios (aORs) of dyslipidemia among those who snored rarely, occasionally, and frequently were 1.1 (95% confidence interval [CI], 1.02–1.18), 1.23 (95% CI, 1.10–1.38), and 1.43 (95% CI, 1.29–1.58), respectively, compared with that among those who never snored. In addition, age and snoring frequency showed a correlation (*P* = 0.02). Sensitivity analysis demonstrated that frequent snoring was significantly associated with lipid levels (all P ≤ 0.01 for linear trend), including increased low-density lipoprotein cholesterol (LDL-C) (β = 0.09 mmol/L; 95% CI, 0.02–0.16), triglyceride (TG) (β = 0.18 mmol/L; 95% CI, 0.10–0.26), total cholesterol (TC) (β = 0.11 mmol/L; 95% CI, 0.05–0.16), and decreased high-density lipoprotein cholesterol (HDL-C) (β=-0.04 mmol/L; 95% CI, -0.06–-0.03).

**Conclusions:**

A statistically significant positive association was identified between sleep snoring and dyslipidemia. It was suggested that sleep snoring interventions may reduce the risk of dyslipidemia.

**Supplementary Information:**

The online version contains supplementary material available at 10.1186/s12944-023-01839-7.

## Background

Dyslipidemia is a manifestation of a lipid metabolism disorder and a risk factor for atherosclerotic cardiovascular disease (ASCVD) [1]. Decreasing lipid levels significantly reduces the risk of ASCVD morbidity and mortality [2]. Therefore, it is crucial to identify the risk factors for dyslipidemia for its prevention and treatment.

Snoring, a common human phenomenon, is the vibration of the upper respiratory tract that occurs during sleep and produces noise as the air moves in and out during breathing. Habitual snoring is common in humans [3], although some variation persists in prevalence statistics due to the non-standardized definition of snoring [4]. Snoring can present as an independent manifestation or a major symptom of obstructive sleep apnea (OSA). Moreover, it reduces sleep quality and is associated with several cardiovascular diseases and their risk factors [5]. OSA is strongly associated with metabolic syndrome [6, 7] and lipid metabolism [8, 9]. However, the diagnosis of OSA requires polysomnography with specialized sleep research equipment and evaluation by a professional physician. Conversely, snoring is easier to identify and monitor in daily life. Snoring exacerbates atherosclerosis and vascular remodeling independent of OSA and other cardiovascular risk factors [10, 11]. Simple snoring alone is still significantly associated with metabolic syndrome after controlling for the effects of recurrent apnea and hypoxia [12] Snoring is also associated with dyslipidemia in some individuals [13, 14]. Therefore, further research needs to be conducted to clarify the association between snoring and dyslipidemia in the general population.

No recent large-scale national studies investigate the association between snoring and dyslipidemia. Therefore, we used the National Health and Nutrition Examination Survey (NHANES) database to analyze the association between snoring and dyslipidemia.

## Methods

### Study sources

**Fig. 1:**
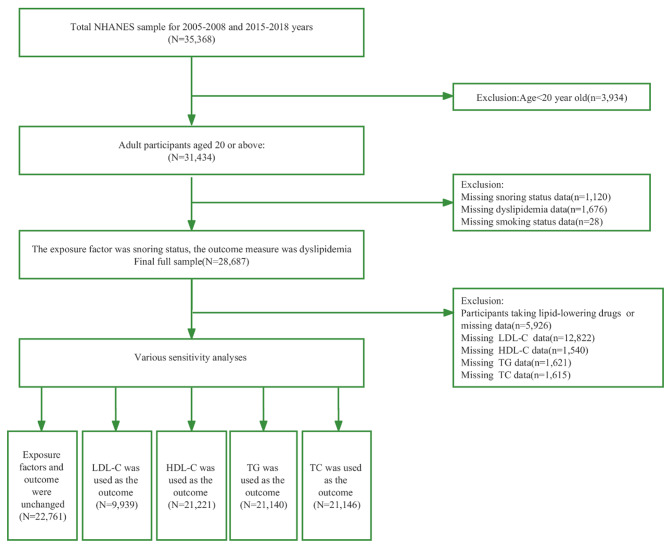
Flow chart showing the number of NHANES participants included in the current analyses

The NHANES is a nationally representative survey of the deinstitutionalized US population, designed to assess the health and nutrition status of adults and children in the United States. These surveys were completed via face-to-face interviews and health examinations at mobile screening centers using a complex, stratified, multistage probabilistic cluster sampling design. The Institutional Review Board of the National Center for Health Statistics approved the study protocol, and all participants provided written informed consent [15]. The current analysis used data from adults aged ≥ 20 years from four cycles conducted from 2005 to 2008 and 2015 to 2018. The inclusion and exclusion criteria for participants are shown in Fig. 1.

### Data collection

Health interviews were conducted at the participants’ homes, and blood collection was performed in mobile examination centers. The collected information included age (20–44, 45–64, ≥ 65 years), sex, race or ethnic group (White, Black, Mexican, or other), education (below high school, high school graduate, or above), ratio of family income to poverty (< 1.3, 1.3–3.49, or ≥ 3.5), and smoking status (never, former, or current smoker). Body mass index (BMI, kg/m^2^) was derived from the reported results of body measurements, and participants were classified into three weight status groups: normal (< 25), overweight (25–30), or obese (≥ 30). Alcohol users were divided into five groups: never, former, mild, moderate, and heavy. Participants were divided into three groups based on their Healthy Eating Index (HEI) scores [16], as follows: “high,” “medium,” and “low.” Physical activity was quartered according to the metabolic equivalent of task scores [17, 18] (Appendix 1).

### Assessment for snoring status, diagnostic criteria for dyslipidemia, and other covariables

#### Snoring status

Snoring status was assessed based on responses to the SLQ030 question on the 2005–2008 and 2015–2018 Sleep Disorders Questionnaires (How often do you snore?). The answers included never, rarely (1–2 nights/week), occasionally (3–4 nights/week), and frequently (≥ 5 nights/week). Missing values for the SLQ030 question were supplemented with responses to the SLQ040 question (How often do you snort or stop breathing?)[19].

#### Dyslipidemia

Dyslipidemia was defined as having any one of the following:

- High TG level: triglyceride (TG) ≥ 150 mg/dl (3.89 mmol/l).
- Hypercholesterolemia: total cholesterol (TC) ≥ 200 mg/dl (5.18 mmol/l), low-density lipoprotein cholesterol (LDL-C),≥130 mg/dl (3.37 mmol/l), and high-density lipoprotein cholesterol (HDL-C) < 40 mg/dl (1.04 mmol/l [males]) and 50 mg/dl (1.30 mmol/l [females])[20].

#### Current alcohol use

Alcohol use was determined as follows: heavy (≥ 3 drinks/day for women, ≥ 4 drinks/day for men, or binge drinking on ≥ 5 days/month), moderate (≥ 2 drinks/day for females, ≥ 3 drinks/day for males, or binge drinking ≥ 2 days/month), mild (the above conditions are not met), former (had ≥ 12 drinks in 1 year and did not drink last year, or did not drink last year but drank ≥ 12 drinks in a lifetime), and never (had < 12 drinks in a lifetime) [21].

#### HEI

The HEI was updated according to the 2015–2020 Dietary Guidelines for Americans [22]. The overall HEI score ranges from 0 to 100, with a higher score indicating a better-quality diet. Scores of > 80, 51–80, and < 50 indicate good, need for improvement, and poor diet, respectively.

### Statistical analyses

Categorical and hierarchical variables were used for statistical analyses, and statistical descriptions are expressed as percentages (95% confidence interval [CI]). The chi-square, Wilcoxon, and Kruskal–Wallis tests were used to analyze the demographic and other characteristics of the participants. The dependent variable was dyslipidemia (yes or no), and the independent variable was snoring status (never, rarely, occasionally, or frequently). Multivariate models included age, sex, BMI, race/ethnicity, ratio of family income to poverty, smoking status, alcohol use, HEI score, and physical activity as covariates. Univariate analysis based on a weighted generalized linear model (GLM) was used to screen variables, which were then included in the multivariate analysis to adjust for confounding factors affecting dyslipidemia. Odds ratios (ORs) with 95% CIs were used to determine the effect size of the comparison. In addition, multivariate logistic regression including product terms was used to explore the possible multiplicative scale interactions between variables. Furthermore, a 2*2 addition model was constructed for statistically significant interaction terms to determine whether the interaction terms had an additive interaction from a biological perspective. Age had three subgroups, and snoring frequency had four subgroups, using the 20–44 age group and never snoring as the respective references, producing six combinations of 2*2 (i.e., six models). Additionally, we calculated the relative excess risk due to interaction (RERI), attributable proportion due to interaction (AP), and synergy index (S). The interaction was visualized by GLM logistic and marginal effect models. Simultaneously, to verify the robustness of the results, lipid profiles from participants with dyslipidemia who did not take lipid-lowering drugs were taken as the outcome variables to conduct sensitivity analysis.

Missing data on snoring as an exposure factor and dyslipidemia as an outcome variable were deleted, with remarkably little missing data on smoking status. For other missing covariates, predictive mean matching was used to perform multiple interpolations for missing data of continuous variables, and missing categorical variable data were considered new subgroups. The association between snoring and dyslipidemia was assessed using GLM with a complete case approach.

All analyses used weighted samples, considering the stratification and clustering of the design and sampling weights estimated as the inverse probability of being selected for the survey, to obtain nationally representative estimates for the United States [23]. To provide estimates for the entire 8 years, a weight variable sample was created by taking one-fourth of the 2-year weight for each person who was sampled in 2005–2005 and 2015–2018.

In the sensitivity analysis, a subset of the population who did not take lipid-lowering medications was selected. First, the exposure factors and outcome variables were unchanged, and the multivariate logistic regression analysis of snoring and dyslipidemia was continued. Second, the exposure factors remained unchanged, and the outcome variables were changed to continuous variable lipid spectrum series values (low-density lipoprotein cholesterol [LDL-C], high-density lipoprotein cholesterol [HDL-C], triglyceride [TG], total cholesterol [TC]), and multifactor linear regression was performed. Two sensitivity analyses were used to further verify the association between snoring and dyslipidemia. The covariates remained constant in all the models. Data analysis was performed using R Statistical Software (version 4.2.0, R Foundation for Statistical Computing, Vienna, Austria), and statistical significance was defined as a two-sided *P* ≤ 0.05.

## Results

### Population characteristics

The 28,687 adult participants who met the inclusion criteria represented a total population of 276,511,553 people in the United States. A description of the study sample is provided in Table 1. The population with varying degrees of snoring accounted for 68.8%. Except for race, the between-group differences were statistically significant. With the increase in snoring frequency, the likelihood of dyslipidemia was higher in the 45–64 age group and obese participants. A description of the study sample with dyslipidemia is provided in Table S1 (Supplementary Appendix) and accounted for 67.18% of the population. Except for sex and HEI score, statistically significant differences were observed between the groups.

**Table 1:** Demographic and other characteristics of adults by percent of snoring status

Characteristic	Snoring status (n = 28,687)				*P* value^*^
	Weighted percent (%) (95% CI)				
	Never (n = 8,948)	Rarely (n = 6,261)	Occasionally (n = 5,196)	Frequently (n = 8,282)	
Dyslipidemia					< 0.001
No	40.82 (39.28–42.36)	36.69 (35.04–38.34)	30.07 (27.89–32.25)	24.83 (23.25–26.41)	
Yes	59.18 (57.64–60.72)	63.31 (61.66–64.96)	69.93 (67.75–72.11)	75.17 (73.59–76.75)	
Age (years)					< 0.001
20–44	52.67 (50.72–54.62)	49.24 (47.22–51.26)	40.63 (38.29–42.96)	38.92 (37.19–40.65)	
45–64	26.84 (25.37–28.32)	35.18 (33.59–36.76)	38.68 (36.85–40.50)	42.72 (40.93–44.51)	
≥65	20.49 (19.17–21.80)	15.58 (14.30–16.86)	20.69 (18.60–22.79)	18.36 (17.07–19.64)	
Sex					< 0.001
Female	61.47 (60.15–62.79)	53.68 (51.95–55.42)	49.34 (47.52–51.15)	40.58 (38.88–42.28)	
Male	38.53 (37.21–39.85)	46.32 (44.58–48.05)	50.66 (48.85–52.48)	59.42 (57.72–61.12)	
BMI					< 0.001
<25	42.86 (41.12–44.60)	32.09 (30.16–34.02)	23.27 (21.86–24.68)	15.14 (13.65–16.63)	
25–29.99	31.11 (29.82–32.40)	34.62 (33.12–36.12)	35.99 (34.15–37.83)	29.74 (28.22–31.26)	
≥30	26.03 (24.62–27.44)	33.29 (31.42–35.16)	40.74 (38.79–42.68)	55.12 (53.35–56.90)	
PIR_group					0.001
<1.3	23.07 (21.57–24.57)	17.02 (15.90–18.14)	17.28 (15.78–18.78)	20.24 (18.72–21.76)	
1.3–3.49	36.16 (34.74–37.59)	34.42 (32.33–36.51)	37.34 (35.06–39.63)	36.44 (34.35–38.54)	
≥3.5	40.77 (38.70–42.84)	48.56 (46.00–51.12)	45.38 (42.67–48.09)	43.32 (40.66–45.97)	
Race or ethnicity					0.06
White	65.61 (62.52–68.69)	66.14 (63.42–68.86)	66.59 (63.48–69.70)	65.16 (61.89–68.44)	
Black	11.87 (10.18–13.57)	10.45 (8.86–12.04)	11.93 (10.21–13.66)	11.03 (9.30–12.77)	
Mexican	7.94 (6.55–9.33)	8.59 (7.07–10.11)	7.93 (6.58–9.28)	9.25 (7.68–10.81)	
Others	14.58 (12.92–16.23)	14.82 (12.99–16.65)	13.54 (11.83–15.26)	14.56 (12.93–16.18)	
Smoking status					< 0.001
Never	60.16 (58.19–62.13)	58.82 (57.00–60.64)	55.19 (53.31–57.06)	48.09 (46.21–49.98)	
Former	21.75 (20.51–22.99)	24.37 (22.87–25.86)	25.59 (24.11–27.07)	28.15 (26.69–29.62)	
Now	18.09 (16.54–19.63)	16.82 (15.57–18.07)	19.22 (17.71–20.73)	23.75 (22.06–25.45)	
Alcohol user					< 0.001
Never	11.77 (10.68–12.85)	8.77 (7.48–10.06)	7.99 (6.88–9.11)	7.26 (6.53–7.99)	
Former	8.54 (7.56–9.52)	6.33 (5.52–7.13)	8.27 (6.98–9.55)	10.15 (8.81–11.49)	
Mild	30.38 (28.61–32.15)	35.82 (33.65–37.99)	35.74 (33.83–37.64)	33.67 (32.24–35.11)	
Moderate	16.66 (15.40–17.93)	17.90 (16.62–19.18)	16.26 (14.93–17.60)	14.52 (13.28–15.77)	
Heavy	17.54 (16.42–18.65)	19.10 (17.43–20.77)	18.17 (16.71–19.63)	21.35 (19.84–22.87)	
Missing	15.11 (13.92–16.30)	12.08 (10.89–13.27)	13.57 (12.32–14.82)	13.04 (11.77–14.31)	
HEI score					< 0.001
Low	48.83 (47.19–50.47)	50.63 (48.54–52.71)	52.87 (50.65–55.09)	54.70 (52.89–56.51)	
Middle	48.71 (47.14–50.28)	47.51 (45.47–49.55)	45.21 (43.03–47.38)	43.75 (41.94–45.56)	
High	2.46 (1.91–3.02)	1.86 (1.45–2.26)	1.92 (1.50–2.35)	1.55 (1.22–1.89)	
Physical activity (MET)					< 0.001
Q1	26.01 (24.67–27.34)	21.97 (20.35–23.60)	25.04 (23.31–26.77)	27.21 (25.76–28.66)	
Q2	25.57 (24.32–26.82)	22.28 (20.79–23.78)	24.83 (23.11–26.55)	23.98 (22.48–25.48)	
Q3	26.48 (25.17–27.80)	28.93 (27.33–30.53)	25.11 (23.75–26.47)	24.36 (22.89–25.83)	
Q4	21.94 (20.60–23.29)	26.82 (24.97–28.66)	25.02 (23.43–26.62)	24.45 (22.75–26.16)	

HEI, Healthy Eating Index; PIR, Ratio of family income to poverty;BMI, body mass index; OR, odds ratio; CI, confidence interval; NHANES, National Health and Nutrition Examination Survey; MET, metabolic equivalent of task

^*^The Kruskal–Wallis test was used for comparison between groups

### Association between snoring and dyslipidemia

Unadjusted univariate logistic regression analysis showed a significant positive association between snoring frequency and dyslipidemia; with an increase in snoring frequency, aORs of dyslipidemia increased correspondingly. Moreover, the in-group comparison and trend test results were statistically significant. All covariables, except sex and HEI score, showed significant associations (Table S2).

**Fig. 2:**
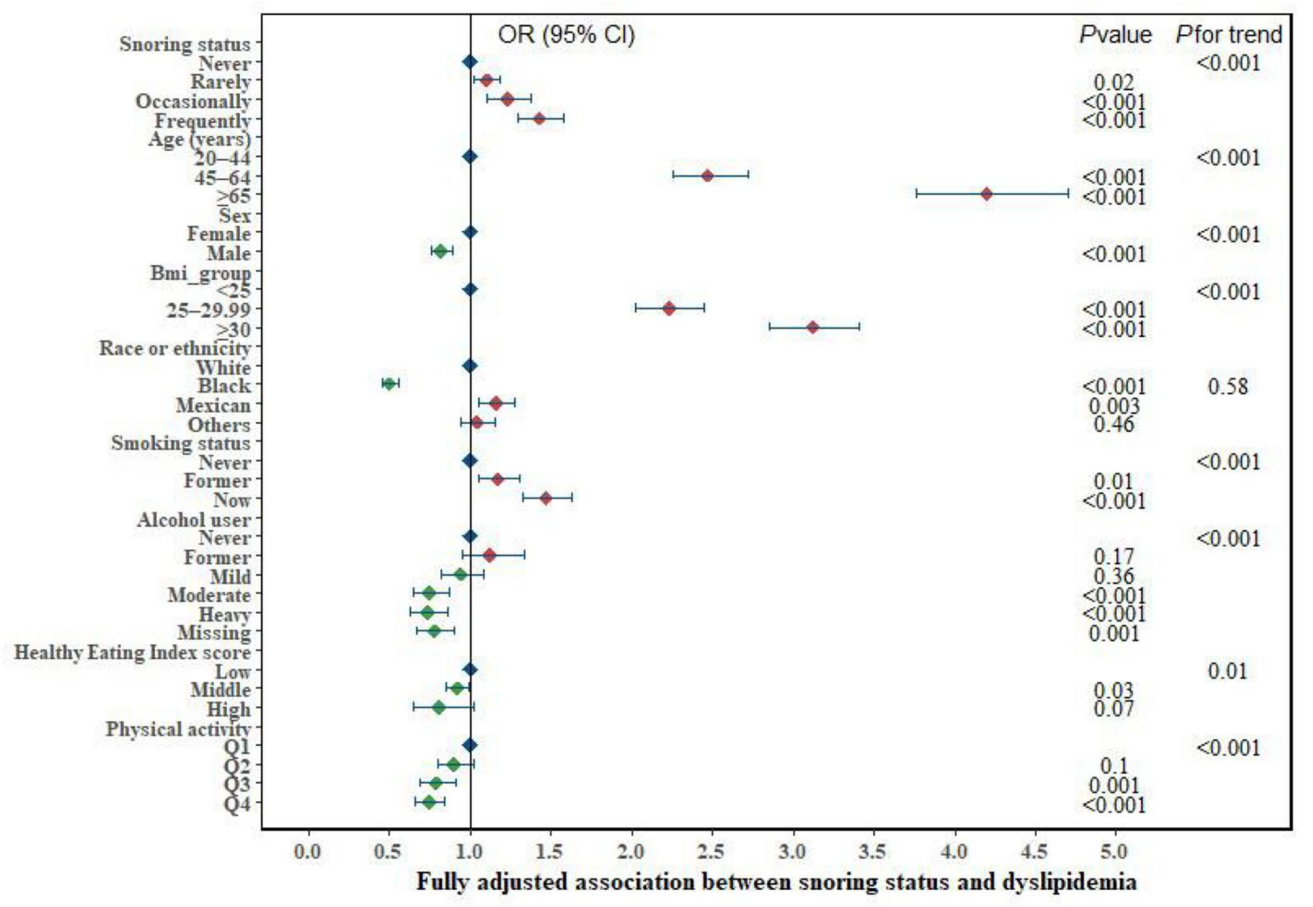
Multivariate analyses of the association between snoring and dyslipidemia. (Abbreviations: BMI, body mass index; CI, confidence interval; OR, odds ratio. Adjusted variables: age, sex, BMI, race or ethnicity, smoking status, alcohol use status, Healthy Eating Index score, and physical activity)

All the results of multiple models with different variables suggested significant associations between snoring frequency and dyslipidemia. The fully adjusted model, adjusted for age, sex, race or ethnicity, smoking status, alcohol use, HEI score, and physical activity status, showed a significant positive association between snoring frequency and dyslipidemia. Adjusted ORs of dyslipidemia among individuals who snored rarely, occasionally, and frequently were 1.1 (95% CI, 1.02–1.18), 1.23 (95% CI, 1.10–1.38), and 1.43 (95% CI, 1.29–1.58), respectively, compared with those who never snored (*P* < 0.001 for linear trend) (Table 2). In addition, other covariates, such as age, sex, body weight, and smoking status, were positively associated with dyslipidemia (all P < 0.001 for linear trend), with age and BMI having the highest ORs for dyslipidemia. Alcohol status, HEI score, and physical activity were negatively associated with dyslipidemia (*P* < 0.001 for linear trend). Moderate alcohol use or above, moderately healthy diet or above, and moderate or more physical activity reduce the OR of dyslipidemia. Only race or ethnicity was not associated with dyslipidemia (Fig. 2 and Table S3).

**Table 2:** Multiple model analyses of the association between snoring and dyslipidemia

Exposure	Crude model	Model 1	Model 2
	OR (95% CI) *P*	OR (95% CI) *P*	OR (95% CI) *P*
Snoring status			
**Never**	**Ref**	**Ref**	**Ref**
Rarely	1.19 (1.10–1.28) < 0.001	1.20 (1.11–1.30) < 0.001	1.10 (1.02–1.18) 0.03
Occasionally	1.60 (1.45–1.77) < 0.001	1.51 (1.36–1.68) < 0.001	1.23 (1.10–1.38) < 0.001
Frequently	2.09 (1.89–2.31) < 0.001	2.00 (1.80–2.22) < 0.001	1.43 (1.29–1.58) < 0.001
*P* for trend (character2integer)	< 0.001	< 0.001	< 0.001

Crude model: No adjustments at all

Model 1: adjusted for age, sex, BMI, ratio of family income to poverty, and race or ethnicity

Model 2: adjusted for age, sex, BMI, race or ethnicity, smoking status, alcohol use status, Healthy Eating Index score, and physical activity

Abbreviations: OR, odds ratio; 95% CI, 95% confidence interval; BMI, body mass index

### Stratified and interaction analyses of the association between snoring and dyslipidemia among different subgroups of different covariates

**Fig. 3:**
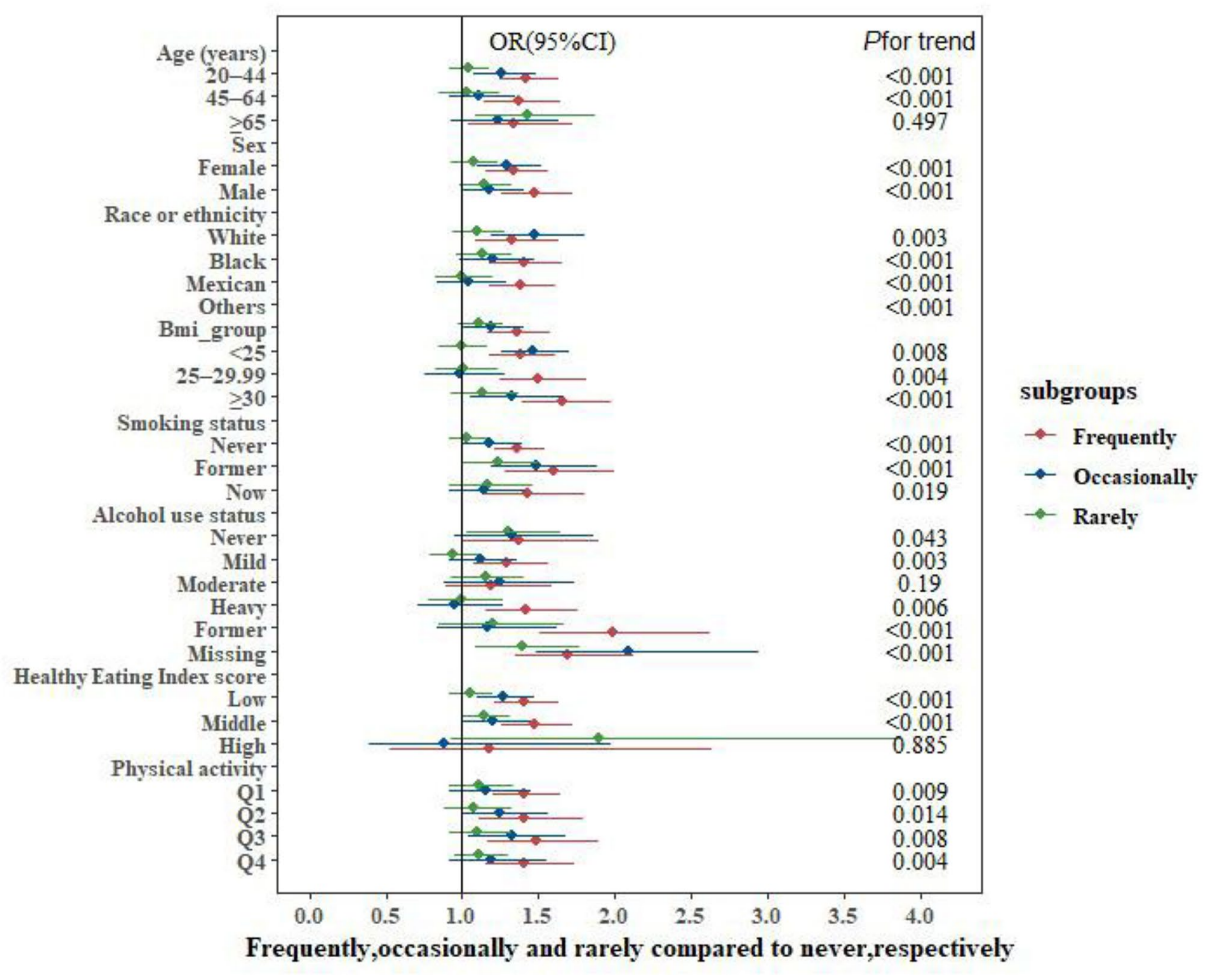
Multivariate analyses of the association between snoring and dyslipidemia stratified by covariates. Abbreviations: BMI, body mass index; OR, odds ratio; CI, confidence interval. Adjusted variables: age, sex, BMI, race or ethnicity, smoking status, alcohol use status, Healthy Eating Index score, and physical activity

Most subgroups of covariates showed trends of positive association with dyslipidemia as snoring frequency increased, compared with non-snorers. Heterogeneity was not shown in subgroups of age, sex, race or ethnicity, smoking status, different intensities of physical activity, and other variables, all of which showed significant associations between frequent snoring and dyslipidemia (all *P* < 0.001 for linear trend). No significant association was identified between snoring frequency and dyslipidemia in the healthier diet quality subgroup of the HEI score and in never and moderate alcohol users. In addition, a baseline comparison between the missing alcohol use and non-missing groups indicated significantly more participants aged ≥ 65 years in the missing data group than those in the non-missing group, and this age group was significantly positively associated with dyslipidemia. This can explain the significant association between alcohol use and dyslipidemia in the missing data group (Fig. 3 and Table S4-S5).

**Fig. 4:**
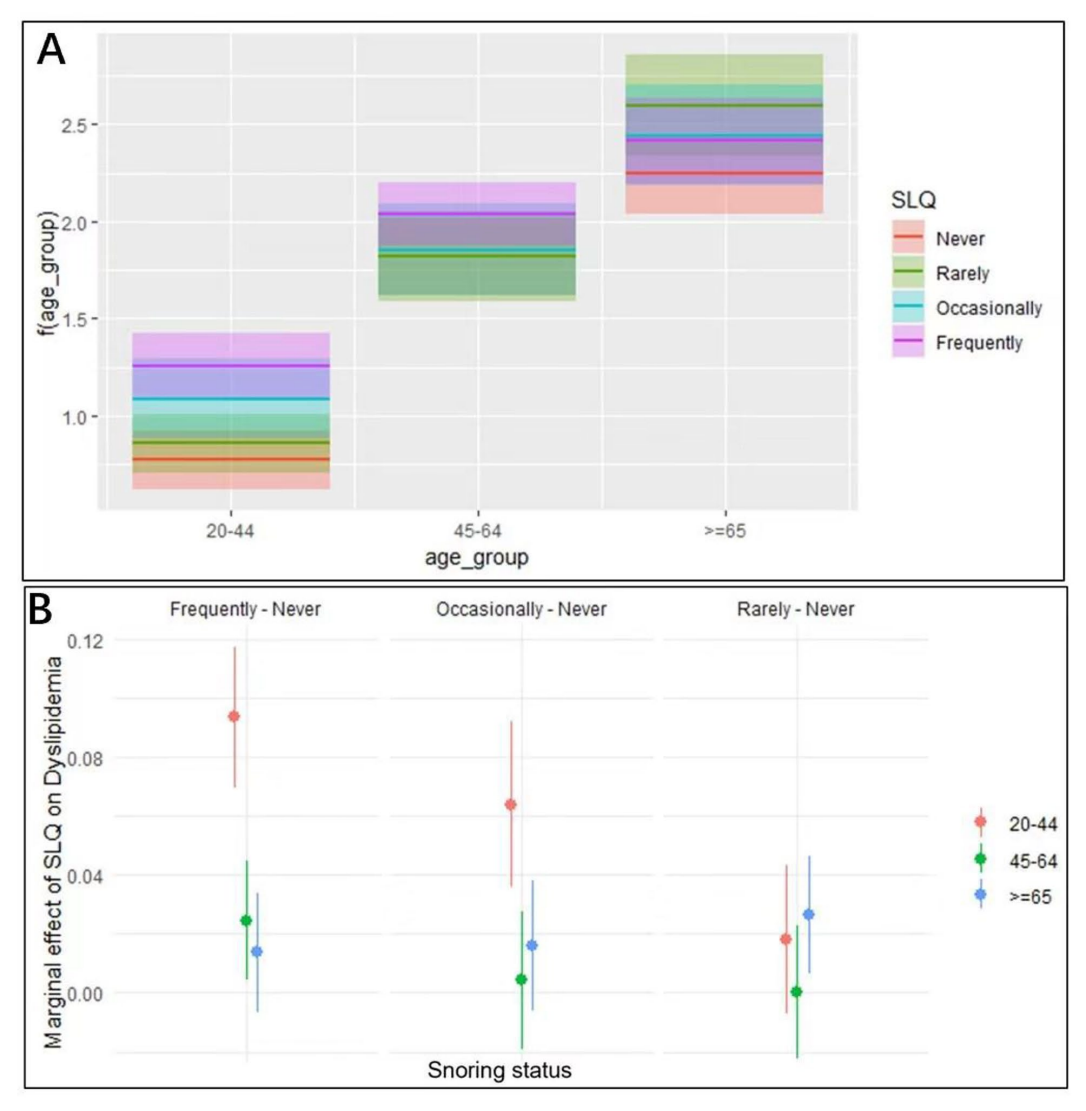
Visualization of interaction between snoring and dyslipidemia. (Abbreviations: SLQ, snoring status. Adjusted variables: snoring status, age, sex, BMI, race or ethnicity, smoking status, alcohol use status, Healthy Eating Index score, and physical activity. The images showed changes in the effect of snoring frequency on dyslipidemia with age. A) shows segmental fitting of snoring status and interaction terms for each age group using logistic regression, and B) visualizes the interaction between snoring status and age groups using the marginal effects model)

Fully adjusted multivariate regression with multiplicative scale interaction terms showed that the association between snoring frequency and dyslipidemia was significantly modified by age (*P* = 0.02), and aORs of dyslipidemia did not increase with the frequency of snoring in the different age groups. In contrast, the effect of snoring frequency on dyslipidemia was gradually weakened by aging, showing an antagonistic effect. The aORs of dyslipidemia were 0.76 (95% CI, 0.59–0.97) for occasional snoring, 0.77 (95% CI, 0.62–0.97) for frequent snoring in the 45–64 years age group, and 0.73 (95% CI, 0.57–0.95) for frequent snoring in people aged ≥ 65 years (Fig. 4 and Table S6). No interaction was observed between the other covariates and snoring status(*P* > 0.05).

The additive interaction of different age groups and the snoring frequency on blood lipid showed that Model 4, with those aged ≥ 65 years and rarely snoring, was the only interaction item that maintained significance among the six models (RERI = 1.81 [95% CI, 0.18–3.45], AP = 0.28 [95% CI, 0.08–0.48], and S = 1.50 [95% CI, 0.97–2.31]). This indicated a significant synergistic effect between rarely snoring and age ≥ 65 years. When both were present, the risk of dyslipidemia increased by 81%, and 28% of the combined interaction in people with dyslipidemia was attributable to age ≥ 65 years and rarely snoring (Table S7 and Figure S1). The multiplicative and additive interactions rendered the same results.

### Sensitivity analysis

In total, 24 participants with missing lipid-lowering medication data and 5,902 participants taking lipid-lowering medications were excluded, reducing the total study population to 22,761, including 13,357 participants (58.68%) with dyslipidemia. Multivariate logistic regression analysis of snoring frequency and dyslipidemia showed that snoring frequency was significantly positively associated with dyslipidemia (*P* < 0.001 for linear trend) (Table S8).

**Fig. 5:**
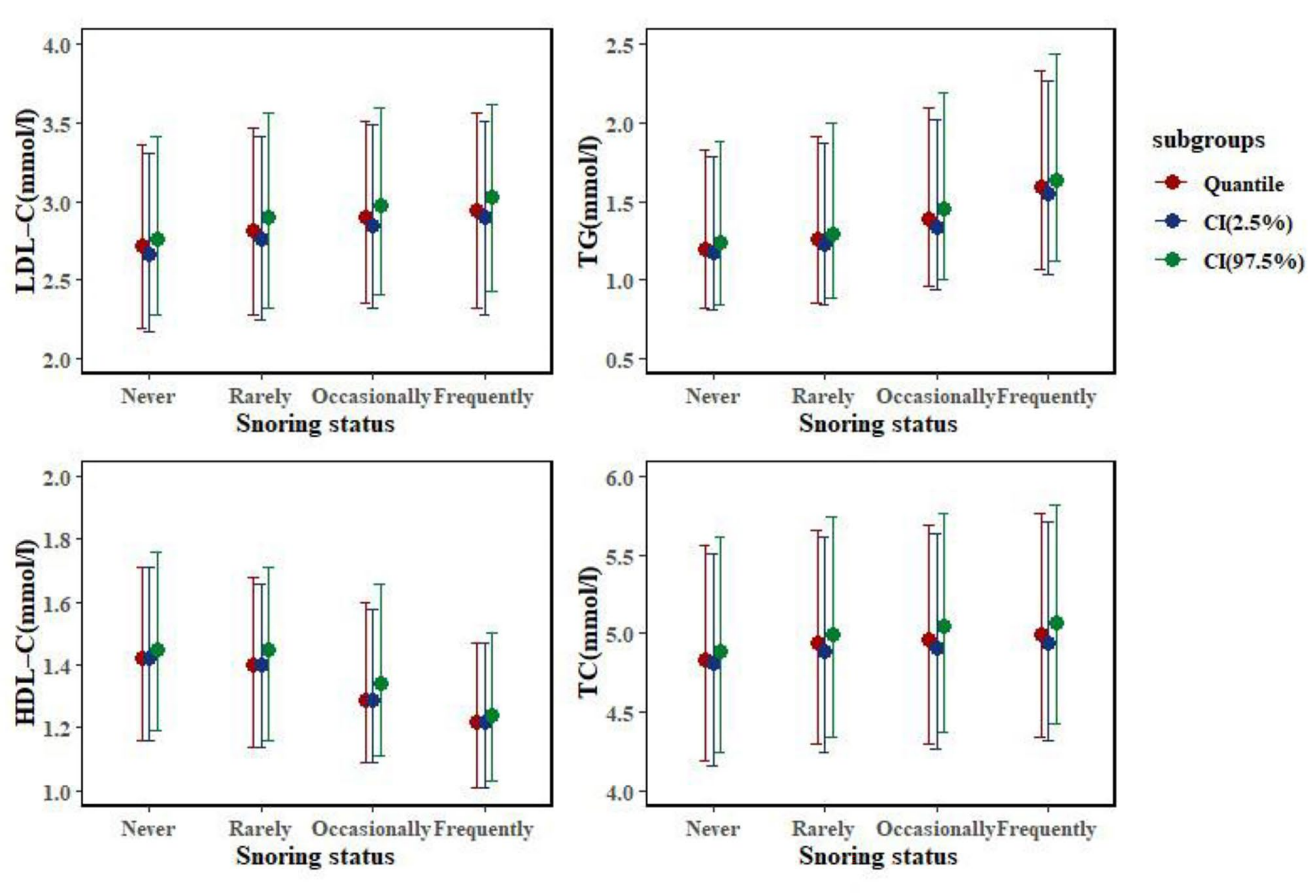
Quartile comparison of blood lipid profile levels among subgroups of snoring state. (Abbreviations: CI, confidence interval; LDL-C, low-density lipoprotein cholesterol; TG, triglyceride; HDL-C, high-density lipoprotein cholesterol; TC, total cholesterol. Error bars indicate 95% confidence intervals. Adjusted variables: snoring status, age, sex, BMI, race or ethnicity, smoking status, alcohol use status, Healthy Eating Index score, and physical activity)

In another sensitivity analysis, with LDL-C, TG, TC, and HDL-C levels as outcome indices and snoring status as an exposure factor, multivariate linear regression was continued. The results of full correction showed that the frequent snoring group had significant differences in the above four indicators, compared with the never-snoring group. Frequent snorers had significantly increased levels of LDL-C (β = 0.09 mmol/L, [95% CI, 0.02–0.16]) (Table S9), TG (β = 0.18 mmol/L [95% CI, 0.10–0.26]) (Table S10), and TC (β = 0.11 mmol/L [95% CI, 0.05–0.16]) (Table S11), along with significantly decreased HDL-C levels (β= -0.04 mmol/L [95% CI, -0.06–-0.03]) (Table S12) with *P* < 0.05. The results of the multivariate analysis of each lipid profile index are shown in Fig. 5.

Overall, the results of our sensitivity analyses were consistent with those of the primary analysis.

## Discussion

According to a large national sample of adults in the United States who participated in the NHANES survey, after adjusting for several potential confounding factors, a significant positive association between snoring and dyslipidemia was identified. This is similar to the results of previous studies.

The stratified analysis showed homogeneity among subgroups, except for alcohol consumption and HEI scores. Frequent snorers were more likely to have increased ORs of dyslipidemia in men, those who were overweight and obese, Mexicans and other ethnic groups, and smokers. Moreover, a healthy diet eliminated the association between snoring and dyslipidemia. Interestingly, our study showed that frequent snoring was not significantly associated with dyslipidemia in moderate drinkers. This is concordant with previous research. The health effects of alcohol may vary depending on the consumed amounts. Low to moderate drinkers have a lower risk of cardiovascular disease than lifetime abstainers, while heavy drinkers have the highest risk [24]. A meta-analysis showed that a lifestyle encompassing moderate alcohol consumption, physical activity, healthy diet, and not smoking reduced the risk of all-cause death by 66% [25].

Only age and snoring status showing potential interactions. The association between frequent snoring and blood lipids weakened with age, but significant synergistic effects were observed between those aged ≥ 65 years old and those who rarely snore. The results of these interactions were reflected comprehensively in the stratified analysis of age. Compared with young people, elderly individuals have more factors that contribute to abnormal blood lipids, such as relatively decreased energy consumption, decreased activity of LDL receptors, and decreased LDL turnover rate. In addition, elderly individuals have specific sleep characteristics, such as reduced sleep time, slow wave sleep, and fragmented sleep. We speculate that these factors may lead to different effects of snoring on blood lipid abnormalities in elderly individuals compared with young people; however, there is currently no evidence to support this view. In middle-aged and elderly individuals, self-reported snoring was significantly correlated with carotid atherosclerosis[26]. Therefore, the impact of snoring on blood lipids in middle-aged and elderly individuals still needs attention. The potential effect of age on the relationship between snoring and blood lipid abnormalities deserves further observation and study.

Various studies have explored the association between snoring and dyslipidemia. A study conducted in Japan investigated the association between snoring and lifestyle-related diseases in an occupational population. The results showed that snoring was an independent comorbid factor of dyslipidemia in men aged 20–59 years [13], which was slightly different from our results. A study conducted in China investigated patients in a rural area [14]. The multistage, stratified, random cluster sampling scheme showed that snoring intensity was significantly associated with dyslipidemia in adults with BMI values of ≥ 25 kg/m^2^, but not in those with BMI values of < 25 kg/m^2^ [14]. This is consistent with our findings. Our study has some advantages over these studies. We adopted the NHANES database, which increased the applicability of the results. Simultaneously, considering that taking lipid-lowering medications can affect lipid levels, and some patients without dyslipidemia but with ASCVD or stroke require lipid-lowering medications for secondary prevention, we performed a sensitivity analysis to confirm the stability of the results after excluding individuals taking lipid-lowering medications, The results confirmed the association between snoring and dyslipidemia.

Controversies remain regarding past changes in lipid profiling. A Chinese study showed that self-reported snoring was significantly associated with high TC and LDL-C, rather than low HDL-C and high TG [14]. Additionally, another study showed that compared with non-snorers, simple snorers had higher TC, TG, LDL-C, and apolipoprotein B, lower apolipoprotein A-I, and a higher prevalence of high TG [12]. A Korean study that focused solely on TG and HDL-C showed that snoring more than six times per week was associated with an increased risk of high TG and low HDL-C in both men and women [27]. Our sensitivity analysis results showed that with an increase in snoring frequency, LDL-C, TC, and TG significantly increased, whereas HDL-C significantly decreased. After adjusting for confounding factors, the trend persisted; however, the differences between snoring frequency and lipid levels gradually narrowed, with only frequent snorers remaining significantly different. Therefore, it is hypothesized that an excessive frequency of snoring may affect lipid levels, and the threshold may be five or more instances of snoring per week. Current clinical studies have different definitions of habitual snoring and snoring frequency [28]. Therefore, large-scale prospective clinical studies are required for further exploration and verification.

The mechanism by which snoring is associated with dyslipidemia remains unclear. Animal experiments have shown that snoring may induce carotid endothelial dysfunction through vibration, resulting in local carotid atherosclerosis [29]. However, snoring has been associated with an increased risk of cardiovascular events [30]. Therefore, the damage caused by snoring on the human body is not localized and has a systemic effect. Individuals who snore often have apnea, which leads to hypopnea and chronic intermittent hypoxia (CIH). CIH increases lipolysis and transport of adipose tissue to the liver and promotes the biosynthesis of cholesterol and TGs [31]. The clearance of lipoproteins is also delayed by the inhibition of lipoprotein lipase in adipose tissues [32]. Hypoxia affects lipid function through oxidative stress and causes lipid peroxidation, producing more oxidized LDL-C [33]. In addition, a genetic association between snoring and lipid metabolism was found, with DLEU7 and MSRB3 as the main genes [3]. DLEU7 is associated with cardiovascular disease and systolic blood pressure, whereas MSRB3 plays a relevant role in protein and lipid metabolism.

Our results support the importance of implementing appropriate snoring interventions. Although snoring is not considered a disease, its emerging association with arteriosclerosis and metabolic syndrome should be seriously considered. Thus, snoring can be effectively intervened. The guidelines for snoring treatment recommend that sleep physicians prescribe oral appliances, rather than no therapy, for adult patients who fail conservative measures and request further treatment for primary snoring without OSA [34]. Appropriate treatment improves patients’ quality of life and health-related outcomes [34].

Our study has some limitations. First, self-reporting of snoring status introduces potential misclassification bias. Second, despite adjusting for nine confounding factors, other factors may still affect the accuracy of the results. Third, we reported the prevalence of dyslipidemia in the general population aged ≥ 20 years, but this is only a range and cannot report precise values. Fourth, we reported a prevalence range of dyslipidemia in the general population aged ≥ 20 years. In our study, 67.18% of the patients had dyslipidemia by laboratory tests or were treated with lipid-lowering medications. This figure is higher than the actual prevalence because it includes individuals who do not have dyslipidemia but are taking lipid-lowering medications as a secondary prevention. Owing to the inability to accurately distinguish the above populations, we excluded all participants taking lipid-lowering medications and reported a prevalence of 58.68%. Therefore, we estimated that the prevalence of dyslipidemia ranges from 58.68 to 67.18%. Fifth, it is possible that only a small part of the snoring population in this study were simple snorers, and most had OSA: the relationship between OSA and metabolic diseases such as dyslipidemia has been relatively clear. In the end, the database we used was a cross-sectional study; thus, our results can be used only to assess association, not to determine causation.

## Conclusions

In this analysis of the NHANES database, snoring was significantly positively associated with dyslipidemia. Our results support the importance of implementing appropriate interventions for snoring, avoiding excessive alcohol consumption, and maintaining a healthy diet and weight.

## Electronic supplementary material

Below is the link to the electronic supplementary material.

Supplementary Material 1

## Data Availability

The datasets analyzed during the current study are available in the NHANES [https://www.cdc.gov/nchs/nhanes/index.htm].

## List of abbreviations

aOR: adjusted odds ratio
AP: attributable proportion due to interaction
ASCVD: Atherosclerotic cardiovascular disease
BMI: body mass index
CI: confidence interval
CIH: chronic intermittent hypoxia
GLM: generalized linear model
HDL-C: high-density lipoprotein cholesterol
HEI: Healthy Eating Index
LDL-C: low-density lipoprotein cholesterol
NHANES: National Health and Nutrition Examination Survey
OR: odds ratio
OSA: obstructive sleep apnea
RERI: relative excess risk due to interaction
S: synergy index
TC: total cholesterol
TG: triglyceride
