# Supplementary material for: Positive correlation between snoring and dyslipidemia in adults: results from NHANES
Source: Lipids Health Dis. 2023 Jun 16;22:73. doi: 10.1186/s12944-023-01839-7 (PMC10276450; doi:10.1186/s12944-023-01839-7)
Supplement: Supplementary file 1 — Supplementary Material 1 [file 12944_2023_1839_MOESM1_ESM.docx]

**SUPPLEMENTAL MATERIALS**

[**Contents**](javascript:;)

**Tables**

**Table S1 Demographic and other characteristics of adults in the 2005-2008 and 2015-2018 NHANES by percent of dyslipidemia**

**Table S2 Univariate analysis of the correlation between snoring status, demographic characteristics and dyslipidemia**

**Table S3 Fully adjusted multivariate analysis of the correlation between snoring and dyslipidemia**

**Table S4** **Multivariate analysis of the association between snoring and dyslipidemia stratified by covariates**

**Table S5 Baseline characteristics of missing and non-missing data on alcohol use status**

**Table S6 Interaction between snoring and dyslipidemia in full adjusted multivariate analysis**

**Table S7 Interactive effect analysis of age groups and snoring severity**

**Table S****8 Multiple model analyses of the correlation between snoring and dyslipidemia in non-taking participants**

**Table S9 Multiple model analyses of the correlation between snoring status and LDL-C in non-taking participants**

**Table S10 Multiple model analyses of the correlation between snoring status and TG in non-taking participants**

**Table S11** **Multiple model analyses of the correlation between snoring status and TC in non-taking participants**

**Table S12 Multiple model analyses of the correlation between snoring status and HDL-C in non-taking participants**

**Figures**

**Figure. S1 Visualization of the interaction between age and snoring on dyslipidemia**

**Table S1** Demographic and other characteristics of adults in the 2005-2008 and 2015-2018 NHANES survey by percent of dyslipidemia

| Characteristic | Without Dyslipidemia(n=9414) | With Dyslipidemia(n=19273) | *P* value* |
| --- | --- | --- | --- |
|  | Weighted percent(%) (95%CI） | Weighted percent(%) (95%CI） |  |
| Snoring status |  |  | < 0.001 |
| Never | 37.01(35.65–38.37) | 26.95(25.77–28.13) |  |
| Rarely | 25.97(24.81–27.13) | 22.51(21.70–23.33) |  |
| Occasionally | 16.35(15.36–17.34) | 19.10(18.16–20.03) |  |
| Frequently | 20.67(19.39–21.95) | 31.43(30.32–32.55) |  |
| Age (years) |  |  | < 0.001 |
| 20-44 | 64.88(63.28–66.47) | 36.28(34.89–37.66) |  |
| 45-64 | 26.02(24.66–27.38) | 40.09(38.94–41.25) |  |
| ≥65 | 9.10(8.09–10.11) | 23.63(22.41–24.85) |  |
| Sex |  |  | 0.06 |
| Female | 50.50(49.05–51.95) | 52.16(51.44–52.88) |  |
| Male | 49.50(48.05–50.95) | 47.84(47.12–48.56) |  |
| BMI |  |  | < 0.001 |
| <25 | 43.73(42.25–45.20) | 21.65(20.58–22.72) |  |
| 25-29.99 | 28.98(27.70–30.26) | 34.19(33.21–35.17) |  |
| ≥30 | 27.29(25.97–28.61) | 44.16(42.71–45.62) |  |
| Poverty income to ratio (PIR) |  |  | 0.02 |
| <1.3 | 21.00(19.60–22.39) | 19.20(18.10–20.29) |  |
| 1.3-3.49 | 35.79(34.09–37.49) | 36.17(34.78–37.56) |  |
| ≥3.5 | 43.21(41.01–45.41) | 44.64(42.57–46.70) |  |
| Race or ethnicity |  |  | < 0.001 |
| White | 61.00(57.96–64.03) | 68.20(65.45–70.94) |  |
| Black | 15.62(13.60–17.65) | 9.15(7.74–10.56) |  |
| Maxican | 8.27(6.88–9.67) | 8.55(7.15–9.95) |  |
| Other | 15.11(13.36–16.86) | 14.11(12.71–15.50) |  |
| Smoking status |  |  | < 0.001 |
| Never | 60.30(58.54–62.05) | 53.21(51.93–54.49) |  |
| Former | 20.16(18.94–21.37) | 27.21(26.23–28.19) |  |
| Now | 19.55(18.09–21.00) | 19.58(18.54–20.62) |  |
| Alcohol user |  |  | < 0.001 |
| Never | 8.67(7.67– 9.67) | 9.34(8.64–10.05) |  |
| Former | 5.63(4.94– 6.32) | 9.81(8.84–10.78) |  |
| Mild | 30.68(29.04–32.32) | 35.00(33.58–36.42) |  |
| Moderate | 18.69(17.57–19.81) | 15.08(14.34–15.83) |  |
| Heavy | 22.87(21.35–24.39) | 17.18(16.20–18.17) |  |
| Missing | 13.46(12.42–14.50) | 13.58(12.62–14.54) |  |
| Healthy Eating Index |  |  | 0.76 |
| Low | 51.73(49.94–53.53) | 51.57(50.03–53.11) |  |
| Middle | 46.20(44.43–47.97) | 46.51(45.02–48.01) |  |
| High | 2.07(1.63–2.51) | 1.92(1.65–2.19) |  |
| Physical activity (MET) |  |  | < 0.001 |
| Q1 | 21.17(19.98–22.37) | 27.24(26.00–28.48) |  |
| Q2 | 22.40(20.92–23.88) | 25.12(24.22–26.03) |  |
| Q3 | 28.20(26.66–29.74) | 25.22(24.32–26.13) |  |
| Q4 | 28.22(26.60–29.85) | 22.41(21.25–23.57) |  |

Abbreviations: BMI, body mass index; CI, confidence interval; NHANES, National Health and Nutrition Examination Survey. MET, metabolic equivalent of task.

*: The chi-square test, Wilcoxon test were used to analyze was used for comparison between groups.

**Table S2** Univariate analysis of the correlation between snoring status and other covariables and dyslipidemia

| **Character** | **Estimate** | **Std. Error** | **OR(95% CI)** | ***P* value** |  |
| --- | --- | --- | --- | --- | --- |
| Snoring status |  |  |  |  |  |
| Never | Ref | Ref | Ref | Ref |  |
| Rarely | 0.17 | 0.04 | 1.19(1.10–1.28) | <0.001 |  |
| Occasionally | 0.47 | 0.05 | 1.60(1.45–1.77) | <0.001 |  |
| Frequently | 0.74 | 0.05 | 2.09(1.89–2.31) | <0.001 |  |
| Age (years) |  |  |  |  |  |
| 20-44 | Ref | Ref | Ref | Ref |  |
| 45-64 | 1.01 | 0.05 | 2.76(2.51–3.03) | <0.001 |  |
| ≥65 | 1.54 | 0.05 | 4.64(4.18–5.16) | <0.001 |  |
| Sex |  |  |  |  |  |
| Female | Ref | Ref | Ref | Ref |  |
| Male | -0.07 | 0.04 | 0.94(0.87–1.00) | 0.06 |  |
| BMI |  |  |  |  |  |
| <25 | Ref | Ref | Ref | Ref |  |
| 25-29.99 | 0.87 | 0.05 | 2.38(2.17–2.61) | <0.001 |  |
| ≥30 | 1.18 | 0.04 | 3.27(3.00–3.56) | <0.001 |  |
| Ratio of family income to poverty |  |  |  |  |  |
| <1.3 | Ref | Ref | Ref | Ref |  |
| 1.3-3.49 | 0.1 | 0.04 | 1.11(1.02–1.20) | 0.02 |  |
| ≥3.5 | 0.12 | 0.05 | 1.13(1.03–1.24) | 0.01 |  |
| Race or ethnicity |  |  |  |  |  |
| White | Ref | Ref | Ref | Ref |  |
| Black | -0.65 | 0.05 | 0.52(0.47–0.58) | <0.001 |  |
| Mexican | -0.08 | 0.05 | 0.92(0.84–1.02) | 0.11 |  |
| Others | -0.18 | 0.05 | 0.84(0.75–0.92) | <0.001 |  |
| Smoking status |  |  |  |  |  |
| Never | Ref | Ref | Ref | Ref |  |
| Former | 0.42 | 0.05 | 1.53(1.39–1.68) | <0.001 |  |
| Now | 0.13 | 0.05 | 1.14(1.04–1.24) | 0.01 |  |
| Alcohol user |  |  |  |  |  |
| Never | Ref | Ref | Ref | Ref |  |
| Former | 0.48 | 0.08 | 1.62(1.38–1.89) | <0.001 |  |
| Mild | 0.06 | 0.07 | 1.06(0.93–1.21) | 0.4 |  |
| Moderate | -0.29 | 0.07 | 0.75(0.65–0.87) | <0.001 |  |
| Heavy | -0.36 | 0.07 | 0.70(0.61–0.80) | <0.001 |  |
| Missing | -0.07 | 0.07 | 0.94(0.82–1.07) | 0.33 |  |
| Healthy Eating Index score | |  |  |  |  |
| Low | Ref | Ref | Ref | Ref |  |
| Middle | 0.01 | 0.04 | 1.01(0.94–1.09) | 0.79 |  |
| High | -0.07 | 0.11 | 0.93(0.75–1.15) | 0.49 |  |
| Physical activity (MET) |  |  |  |  |  |
| Q1 | Ref | Ref | Ref | Ref |  |
| Q2 | -0.14 | 0.05 | 0.87(0.79–0.97) | 0.01 |  |
| Q3 | -0.36 | 0.06 | 0.70(0.62–0.79) | <0.001 |  |
| Q4 | -0.48 | 0.05 | 0.62(0.55–0.69) | <0.001 |  |

Abbreviations: BMI, body mass index; OR, odds ratio; CI, confidence interval; MET, metabolic equivalent of task.

**Table S3** Fully adjusted multivariate analysis of the correlation between snoring and dyslipidemia

| Character | Estimate | Std. Error | OR(95% CI) | *P* value | *P* for trend |
| --- | --- | --- | --- | --- | --- |
| Snoring status |  |  |  |  | <0.001 |
| Never | Ref | Ref | Ref | Ref |  |
| Rarely | 0.09 | 0.04 | 1.10(1.02–1.18) | 0.02 |  |
| Occasionally | 0.21 | 0.06 | 1.23(1.10–1.38) | <0.001 |  |
| Frequently | 0.36 | 0.05 | 1.43(1.29–1.58) | <0.001 |  |
| Age (years) |  |  |  |  | <0.001 |
| 20-44 | Ref | Ref | Ref | Ref |  |
| 45-64 | 0.91 | 0.05 | 2.47(2.25–2.72) | <0.001 |  |
| ≥65 | 1.44 | 0.06 | 4.20(3.76–4.70) | <0.001 |  |
| Sex |  |  |  |  | <0.001 |
| Female | Ref | Ref | Ref | Ref |  |
| Male | -0.19 | 0.04 | 0.82(0.76–0.89) | <0.001 |  |
| BMI |  |  |  |  | <0.001 |
| <25 | Ref | Ref | Ref | Ref |  |
| 25-29.99 | 0.8 | 0.05 | 2.23(2.02–2.45) | <0.001 |  |
| ≥30 | 1.14 | 0.05 | 3.12(2.85–3.41) | <0.001 |  |
| Race or ethnicity |  |  |  |  |  |
| White | Ref | Ref | Ref | Ref | 0.58 |
| Black | -0.69 | 0.06 | 0.50(0.45–0.56) | <0.001 |  |
| Mexican | 0.15 | 0.05 | 1.16(1.05–1.27) | 0.003 |  |
| Others | 0.04 | 0.05 | 1.04(0.94–1.15) | 0.46 |  |
| Smoking status |  |  |  |  | <0.001 |
| Never | Ref | Ref | Ref | Ref |  |
| Former | 0.16 | 0.05 | 1.17(1.05–1.30) | 0.01 |  |
| Now | 0.38 | 0.05 | 1.47(1.32–1.63) | <0.001 |  |
| Alcohol user |  |  |  |  | <0.001 |
| Never | Ref | Ref | Ref | Ref |  |
| Former | 0.12 | 0.08 | 1.12(0.95–1.33) | 0.17 |  |
| Mild | -0.06 | 0.07 | 0.94(0.82–1.08) | 0.36 |  |
| Moderate | -0.29 | 0.07 | 0.75(0.65–0.87) | <0.001 |  |
| Heavy | -0.31 | 0.08 | 0.74(0.63–0.86) | <0.001 |  |
| Missing | -0.25 | 0.07 | 0.78(0.67–0.90) | 0.001 |  |
| Healthy Eating Index score |  |  |  |  | 0.009 |
| Low | Ref | Ref | Ref | Ref |  |
| Middle | -0.08 | 0.04 | 0.92(0.85–0.99) | 0.03 |  |
| High | -0.21 | 0.11 | 0.81(0.65–1.02) | 0.07 |  |
| Physical activity (MET) |  |  |  |  | <0.001 |
| Q1 | Ref | Ref | Ref | Ref |  |
| Q2 | -0.1 | 0.06 | 0.90(0.80–1.02) | 0.1 |  |
| Q3 | -0.24 | 0.07 | 0.79(0.69–0.91) | 0.001 |  |
| Q4 | -0.29 | 0.06 | 0.75(0.66–0.84) | <0.001 |  |

Abbreviations: BMI, body mass index; OR, odds ratio; CI, confidence interval; MET, metabolic equivalent of task.

adjusted for age, sex, BMI, race or ethnicity, smoking status, alcohol use status, Healthy Eating Index score, and physical activity。

**Table S4** Multivariate analysis of the association between snoring and dyslipidemia stratified by covariates

| **Characteristic** | Snoring status (n=22802) | | | | | | | *P* for  trend | *P* for  interaction |
| --- | --- | --- | --- | --- | --- | --- | --- | --- | --- |
|  | Weighted percent (%) (95%CI) | | | | | | |  |  |
|  | Never | Rarely | *P* value | Occasionally | *P* value | Frequently | *P* value |  |  |
| Age (years) |  |  |  |  |  |  |  |  | 0.02 |
| 20-44 | Ref | 1.04(0.92–1.18) | 0.52 | 1.26(1.07–1.48) | 0.01 | 1.42(1.24–1.63) | <0.001 | <0.001 |  |
| 45-64 | Ref | 1.03(0.85–1.24) | 0.78 | 1.11(0.91–1.35) | 0.32 | 1.37(1.14–1.64) | <0.001 | <0.001 |  |
| ≥65 | Ref | 1.43(1.09–1.87) | 0.01 | 1.23(0.93–1.63) | 0.14 | 1.34(1.04–1.73) | 0.02 | 0.04 |  |
| Sex |  |  |  |  |  |  |  |  | 0.49 |
| Female | Ref | 1.07(0.93–1.23) | 0.35 | 1.29(1.10–1.52) | 0.002 | 1.34(1.15–1.57) | <0.001 | <0.001 |  |
| Male | Ref | 1.14(0.98–1.33) | 0.09 | 1.18(0.99–1.41) | 0.06 | 1.47(1.26–1.72) | <0.001 | <0.001 |  |
| BMI |  |  |  |  |  |  |  |  | 0.20 |
| <25 | Ref | 1.10(0.94–1.28) | 0.22 | 1.47(1.19–1.81) | <0.001 | 1.33(1.09–1.63) | 0.01 | <0.001 |  |
| 25-29.99 | Ref | 1.13(0.96–1.33) | 0.13 | 1.20(0.98–1.47) | 0.07 | 1.40(1.18–1.66) | <0.001 | <0.001 |  |
| ≥30 | Ref | 0.99(0.82–1.20) | 0.94 | 1.04(0.83–1.29) | 0.73 | 1.38(1.18–1.61) | <0.001 | <0.001 |  |
| Race or ethnicity |  |  |  |  |  |  |  |  | 0.34 |
| White | Ref | 1.11(0.97–1.27) | 0.14 | 1.19(1.01–1.41) | 0.04 | 1.36(1.17–1.58) | <0.001 | <0.001 |  |
| Black | Ref | 1.00(0.85–1.17) | 0.96 | 1.46(1.26–1.70) | <0.001 | 1.38(1.18–1.61) | <0.001 | <0.001 |  |
| Mexican | Ref | 1.01(0.82–1.23) | 0.96 | 0.98(0.75–1.28) | 0.9 | 1.50(1.24–1.82) | <0.001 | <0.001 |  |
| Others | Ref | 1.13(0.93–1.37) | 0.21 | 1.32(1.05–1.67) | 0.02 | 1.66(1.39–1.98) | <0.001 | <0.001 |  |
| Smoking status |  |  |  |  |  |  |  |  | 0.63 |
| Never | Ref | 1.03(0.92–1.16) | 0.56 | 1.18(1.00–1.39) | 0.05 | 1.36(1.21–1.54) | <0.001 | <0.001 |  |
| Former | Ref | 1.23(1.00–1.50) | 0.05 | 1.49(1.19–1.88) | <0.001 | 1.60(1.28–2.00) | <0.001 | <0.001 |  |
| Now | Ref | 1.16(0.92–1.46) | 0.2 | 1.14(0.91–1.42) | 0.25 | 1.43(1.14–1.80) | 0.003 | 0.004 |  |
| Alcohol user |  |  |  |  |  |  |  |  | 0.06 |
| Never | Ref | 1.30(1.03–1.65) | 0.03 | 1.33(0.95–1.86) | 0.1 | 1.37(0.99–1.89) | 0.06 | 0.04 |  |
| Mild | Ref | 0.94(0.79–1.11) | 0.45 | 1.12(0.92–1.36) | 0.25 | 1.29(1.07–1.56) | 0.01 | 0.003 |  |
| Moderate | Ref | 1.15(0.93–1.41) | 0.18 | 1.24(0.88–1.74) | 0.22 | 1.19(0.89–1.59) | 0.23 | 0.19 |  |
| Heavy | Ref | 0.99(0.78–1.27) | 0.96 | 0.95(0.71–1.27) | 0.74 | 1.42(1.15–1.76) | 0.001 | 0.01 |  |
| Former | Ref | 1.20(0.85–1.67) | 0.28 | 1.16(0.83–1.62) | 0.36 | 1.99(1.51–2.62) | <0.001 | <0.001 |  |
| missing | Ref | 1.39(1.09–1.77) | 0.01 | 2.09(1.49–2.94) | <0.001 | 1.69(1.35–2.12) | <0.001 | <0.001 |  |
| Healthy Eating Index score | |  |  |  |  |  |  |  | 0.41 |
| Low | Ref | 1.05(0.91–1.20) | 0.52 | 1.27(1.10–1.47) | 0.002 | 1.40(1.21–1.63) | <0.001 | <0.001 |  |
| Middle | Ref | 1.14(0.99–1.31) | 0.07 | 1.20(1.00–1.45) | 0.05 | 1.47(1.26–1.72) | <0.001 | <0.001 |  |
| High | Ref | 1.90(0.93–3.88) | 0.08 | 0.88(0.39–1.98) | 0.76 | 1.18(0.53–2.64) | 0.68 | 0.95 |  |
| Physical activity（MET） | | |  |  |  |  |  |  | 0.99 |
| Q1 | Ref | 1.11(0.92–1.34) | 0.29 | 1.15(0.92–1.45) | 0.22 | 1.40(1.20–1.64) | <0.001 | <0.001 |  |
| Q2 | Ref | 1.08(0.88–1.32) | 0.48 | 1.25(1.01–1.57) | 0.04 | 1.41(1.11–1.79) | 0.01 | <0.001 |  |
| Q3 | Ref | 1.10(0.92–1.32) | 0.3 | 1.32(1.04–1.68) | 0.02 | 1.48(1.16–1.90) | 0.002 | <0.001 |  |
| Q4 | Ref | 1.11(0.95–1.30) | 0.2 | 1.19(0.92–1.55) | 0.18 | 1.41(1.15–1.74) | 0.001 | 0.003 |  |

Abbreviations: BMI, body mass index; OR, odds ratio; CI, confidence interval; MET, metabolic equivalent of task.

**Table S5** Baseline characteristics of missing and non-missing data on alcohol use status

| Characteristic | Alcohol use status | | *P* value* |
| --- | --- | --- | --- |
|  | Weighted percent (%) (95%CI) | |  |
|  | missing(n=4830) | no missing(n=23857) |  |
| Snoring status |  |  | < 0.001 |
| Never | 33.85(32.15–35.54) | 29.76(28.64–30.88) |  |
| Rarely | 21.12(19.53–22.71) | 24.07(23.18–24.95) |  |
| Occasionally | 18.22(16.59–19.85) | 18.17(17.43–18.91) |  |
| Frequently | 26.81(24.81–28.81) | 28.00(27.04–28.95) |  |
| Age(years) |  |  | < 0.001 |
| 20-44 | 38.22(35.93–40.51) | 47.03(45.53–48.54) |  |
| 45-64 | 34.30(31.82–36.78) | 35.56(34.54–36.58) |  |
| ≥65 | 27.48(25.34–29.62) | 17.41(16.28–18.54) |  |
| Sex |  |  | 0.03 |
| Female | 53.75(51.94–55.56) | 51.27(50.50–52.04) |  |
| Male | 46.25(44.44–48.06) | 48.73(47.96–49.50) |  |
| Race or ethnicity |  |  | < 0.001 |
| White | 57.26(53.75–60.76) | 67.12(64.31–69.94) |  |
| Black | 13.65(11.72–15.59) | 10.95( 9.35–12.55) |  |
| Mexican | 8.41(6.94–9.88) | 8.46(7.07–9.86) |  |
| Other | 20.68(18.40–22.96) | 13.46(12.09–14.84) |  |
| Ratio of family income to poverty |  |  | < 0.001 |
| <1.3 | 25.87(23.94–27.79) | 18.85(17.74–19.95) |  |
| 1.3-3.49 | 39.14(36.35–41.94) | 35.56(34.18–36.93) |  |
| ≥3.5 | 34.99(32.20–37.78) | 45.60(43.56–47.63) |  |
| BMI |  |  | 0.26 |
| <25 | 28.41(26.30–30.51) | 29.13(27.99–30.28) |  |
| 25-29.99 | 34.11(32.31–35.92) | 32.18(31.28–33.09) |  |
| ≥30 | 37.48(35.15–39.81) | 38.68(37.35–40.01) |  |
| Smoking status |  |  | 0.68 |
| Never | 55.31(52.87–57.75) | 55.62(54.38–56.86) |  |
| Former | 25.62(23.48–27.77) | 24.73(23.82–25.64) |  |
| Now | 19.07(17.45–20.68) | 19.65(18.51–20.79) |  |
| Healthy Eating Index score |  |  | 0.72 |
| Low | 51.62(49.27–53.96) | 51.62(50.07–53.18) |  |
| Middle | 46.18(43.80–48.56) | 46.44(44.97–47.92) |  |
| High | 2.21(1.65–2.76) | 1.93(1.63–2.23) |  |
| Physical activity（MET） |  |  | 0.12 |
| Q1 | 24.40(22.57–26.22) | 25.34(24.26–26.41) |  |
| Q2 | 24.60(23.02–26.18) | 24.15(23.23–25.07) |  |
| Q3 | 24.84(23.23–26.46) | 26.44(25.62–27.25) |  |
| Q4 | 26.16(24.31–28.01) | 24.07(22.88–25.27) |  |

Abbreviations: BMI, body mass index; OR, odds ratio; CI, confidence interval; MET, metabolic equivalent of task.

*: The chi-square test– Wilcoxon test 888were used to analyze was used for comparison between groups.

**Table S6** Interaction between snoring and dyslipidemia in full adjusted multivariate analysis

| character | Estimate | Std. Error | OR (95% CI) | *P* value |
| --- | --- | --- | --- | --- |
| Snoring status |  |  |  |  |
| Never | Ref | Ref | Ref | Ref |
| Rarely | 0.08 | 0.06 | 1.09(0.97–1.23) | 0.16 |
| Occasionally | 0.32 | 0.07 | 1.37(1.18–1.59) | <0.001 |
| Frequently | 0.48 | 0.06 | 1.61(1.42–1.83) | <0.001 |
| Age(year) |  |  |  |  |
| 20-44 | Ref | Ref | Ref | Ref |
| 45-64 | 1.05 | 0.08 | 2.84(2.43–3.33) | <0.001 |
| >=65 | 1.47 | 0.09 | 4.36(3.63–5.23) | <0.001 |
| Snoring status:  age group |  |  |  | *P* for interaction |
| Rarely: 45-64 | -0.08 | 0.13 | 0.92(0.71–1.19) | 0.52 |
| Occasionally: 45-64 | -0.28 | 0.13 | 0.76(0.59–0.97) | 0.03 |
| Frequently: 45-64 | -0.26 | 0.11 | 0.77(0.62–0.97) | 0.02 |
| Rarely:≥65 | 0.27 | 0.15 | 1.31(0.96–1.78) | 0.09 |
| Occasionally:≥65 | -0.12 | 0.15 | 0.89(0.66–1.21) | 0.45 |
| Frequently:≥65 | -0.31 | 0.13 | 0.73(0.57–0.95) | 0.02 |

Abbreviations: OR, odds ratio; CI, confidence interval

**Table S7 Interactive effect analysis of age groups and snoring severity**

| Model | Age(y) | Snoring staus | Interaction term | Dyslipidemia/Total(n) | OR(95% CI) | *P* |
| --- | --- | --- | --- | --- | --- | --- |
| Model1 | 20-44 | never snoring | A0B0 | 1909/4184 | ref | ref |
|  | 20-44 | rarely snoring | A0B1 | 1475/2935 | 1.07(0.95,1.20) | 0.28 |
|  | 45-64 | never snoring | A1B0 | 1635/2347 | 2.86(2.44,3.36) | <0.001 |
|  | 45-64 | rarely snoring | A1B1 | 1469/2047 | 2.84(2.38,3.38) | <0.001 |
|  | RERI |  |  |  | -0.10(-0.69,0.50) |  |
|  | AP |  |  |  | -0.03(-0.25,0.18) |  |
|  | S |  |  |  | 0.95(0.72,1.25) |  |
| Model2 | 20-44 | never snoring | A0B0 | 1909/4184 | ref | ref |
|  | 20-44 | Occasionally snoring | A0B1 | 1085/1903 | 1.37(1.17,1.60) | <0.001 |
|  | 45-64 | never snoring | A1B0 | 1635/2347 | 2.84(2.43,3.31) | <0.001 |
|  | 45-64 | Occasionally snoring | A1B1 | 1431/1920 | 2.95(2.44,3.57) | <0.001 |
|  | RERI |  |  |  | -0.26(-0.87,0.35) |  |
|  | AP |  |  |  | -0.09(-0.30,0.13) |  |
|  | S |  |  |  | 1.09(0.65,1.27) |  |
| Model3 | 20-44 | never snoring | A0B0 | 1909/4184 | ref | ref |
|  | 20-44 | Frequently snoring | A0B1 | 1822/2833 | 1.50(1.30,1.73) | <0.001 |
|  | 45-64 | never snoring | A1B0 | 1635/2347 | 2.87(2.46,3.34) | <0.001 |
|  | 45-64 | Frequently snoring | A1B1 | 2761/3501 | 3.37(2.90,3.91) | <0.001 |
|  | RERI |  |  |  | 0.004(-0.57,0.57) |  |
|  | AP |  |  |  | 0.001(-0.17,0.17) |  |
|  | S |  |  |  | 1.00(0.78,1.28) |  |
| Model4 | 20-44 | never snoring | A0B0 | 1909/4184 | ref | ref |
|  | 20-44 | rarely snoring | A0B1 | 1475/2935 | 1.05(0.93,1.19) | 0.4 |
|  | ≥65 | never snoring | A1B0 | 1872/2417 | 4.63(3.77,5.67) | <0.001 |
|  | ≥65 | rarely snoring | A1B1 | 1047/1279 | 6.49(5.01,8.42) | <0.001 |
|  | RERI |  |  |  | 1.81(0.18,3.45) |  |
|  | AP |  |  |  | 0.28(0.08,0.48) |  |
|  | S |  |  |  | 1.49(0.97,2.31) |  |
| Model5 | 20-44 | never snoring | A0B0 | 1909/4184 | ref | ref |
|  | 20-44 | Occasionally snoring | A0B1 | 1085/1903 | 1.36(1.16,1.59) | <0.001 |
|  | ≥65 | never snoring | A1B0 | 1872/2417 | 4.35(3.57,5.31) | <0.001 |
|  | ≥65 | Occasionally snoring | A1B1 | 1143/1373 | 5.24(4.20,6.53) | <0.001 |
|  | RERI |  |  |  | 0.53(-0.84,1.89) |  |
|  | AP |  |  |  | 0.10(-0.14,0.34) |  |
|  | S |  |  |  | 1.14(0.80,1.64) |  |
| Model6 | 20-44 | never snoring | A0B0 | 1909/4184 | ref | ref |
|  | 20-44 | Frequently snoring | A0B1 | 1822/2833 | 1.49(1.30,1.72) | <0.001 |
|  | ≥65 | never snoring | A1B0 | 1872/2417 | 4.47(3.69,5.42) | <0.001 |
|  | ≥65 | Frequently snoring | A1B1 | 1624/1948 | 5.07(4.11,6.26) | <0.001 |
|  | RERI |  |  |  | 0.11(-1.10,1.31) |  |
|  | AP |  |  |  | 0.02(-0.21,0.25) |  |
|  | S |  |  |  | 1.03(0.72,1.47) |  |

Abbreviations: OR, odds ratio; CI, confidence interval

**Table S8** Multiple model analyses of the correlation between snoring and dyslipidemia in non-taking participants

| Exposure | Crude model | Model 1 | Model 2 |
| --- | --- | --- | --- |
|  | OR (95%CI) *P* | OR (95%CI) *P* | OR (95%CI) *P* |
| Snoring status |  |  |  |
| Never | Ref | Ref | Ref |
| Rarely | 1.22(1.13–1.32) <0.001 | 1.20(1.11–1.30) <0.001 | 1.10(1.02–1.19) 0.015 |
| Occasionally | 1.51(1.37–1.67) <0.001 | 1.46(1.32–1.63) <0.001 | 1.20(1.07–1.34) 0.002 |
| Frequently | 1.97(1.78–2.19) <0.001 | 1.93(1.73–2.15) <0.001 | 1.40(1.27–1.56) <0.001 |
| *P* for trend (character2integer) | <0.001 | <0.001 | <0.001 |
| Crude model: No adjustments at all | | | |
| Model 1: adjusted for age, sex, BMI, ratio of family income to poverty, and race or ethnicity | | | |
| Model 2: adjusted for age, sex, BMI, ratio of family income to poverty, race or ethnicity, smoking status, alcohol use status, Healthy Eating Index score, and physical activity  Abbreviations: OR, odds ratio; 95% CI, 95% confidence interval; BMI, body mass index | | | |

**Table S9** Multiple model analyses of the correlation between snoring status and LDL-C in non-taking participants

| Exposure | Crude model | Model 1 | Model 2 |
| --- | --- | --- | --- |
|  | β(95%CI)*P* | β(95%CI)*P* | β(95%CI)*P* |
| Snoring status |  |  |  |
| Never | Ref | Ref | Ref |
| Rarely | 0.10(0.02–0.17) 0.013 | 0.06(-0.01–0.13) 0.08 | 0.05(-0.02– 0.12) 0.17 |
| Occasionally | 0.18(0.12–0.25) <0.001 | 0.12(0.05–0.18) <0.001 | 0.07(0.00– 0.14) 0.05 |
| Frequently | 0.24(0.17–0.30) <0.001 | 0.16(0.10–0.23) <0.001 | 0.09(0.02– 0.16) <0.001 |
| *P* for trend (character2integer) | <0.001 | <0.001 | 0.01 |
| Crude model: No adjustments at all | | | |
| Model 1: adjusted for age, sex, BMI, ratio of family income to poverty, and race or ethnicity | | | |
| Model 2: adjusted for age, sex, BMI, ratio of family income to poverty ,race or ethnicity, smoking status, alcohol use status, Healthy Eating Index score, and physical activity  Abbreviations: LDL-C, low-density lipoprotein cholesterol; β: Coefficient; 95% CI, 95% confidence interval | | | |

**Table S10** Multiple model analyses of the correlation between snoring status and TG in non-taking participants

| Exposure | Crude model | Model 1 | Model 2 |
| --- | --- | --- | --- |
|  | β(95%CI)*P* | β(95%CI)*P* | β(95%CI)*P* |
| Snoring status |  |  |  |
| Never | Ref | Ref | Ref |
| Rarely | 0.09(0.04–0.14) 0.001 | 0.04(-0.01– 0.10) 0.11 | -0.01(-0.06– 0.04) 0.68 |
| Occasionally | 0.22(0.16–0.28) <0.001 | 0.14(0.08– 0.21) <0.001 | 0.01(-0.05– 0.07) 0.71 |
| Frequently | 0.52(0.42–0.62) <0.001 | 0.39(0.30– 0.48) <0.001 | 0.18(0.10– 0.26) <0.001 |
| *P* for trend (character2integer) | <0.001 | <0.001 | <0.001 |
| Crude model: No adjustments at all | | | |
| Model 1: adjusted for age, sex, BMI, ratio of family income to poverty, and race or ethnicity | | | |
| Model 2: adjusted for age, sex, BMI, ratio of family income to poverty, race or ethnicity, smoking status, alcohol use status, Healthy Eating Index score, and physical activity  Abbreviations: TG, triglyceride; β, Coefficient; 95% CI, 95% confidence interval | | | |

**Table S11** Multiple model analyses of the correlation between snoring status and TC in non-taking participants

| Exposure | Crude model | Model 1 | Model 2 |
| --- | --- | --- | --- |
|  | β(95%CI)*P* | β(95%CI)*P* | β(95%CI)*P* |
| Snoring status |  |  |  |
| Never | Ref | Ref | Ref |
| Rarely | 0.08(0.03–0.14) 0.003 | 0.06(0.01– 0.11) 0.03 | 0.04(-0.01– 0.10) 0.11 |
| Occasionally | 0.14(0.09–0.19) <0.001 | 0.09(0.04– 0.14) <0.001 | 0.04(-0.01– 0.10) 0.08 |
| Frequently | 0.22(0.17–0.28) <0.001 | 0.17(0.11– 0.23) <0.001 | 0.11(0.05– 0.16) <0.001 |
| *P* for trend (character2integer) | <0.001 | <0.001 | <0.001 |
| Crude model: No adjustments at all | | | |
| Model 1: adjusted for age, sex, BMI, ratio of family income to poverty, and race or ethnicity | | | |
| Model 2: adjusted for age, sex, BMI, ratio of family income to poverty, race or ethnicity, smoking status, alcohol use status, Healthy Eating Index score, and physical activity  Abbreviations: TC, total cholesterol; β, Coefficient; 95% CI, 95% confidence interval | | | |

**Table S12** Multiple model analyses of the correlation between snoring status and HDL-C in non-taking participants

| Exposure | Crude model | Model 1 | Model 2 |
| --- | --- | --- | --- |
|  | β(95%CI)*P* | β(95%CI)*P* | β(95%CI)*P* |
| Snoring status |  |  |  |
| Never | Ref | Ref | Ref |
| Rarely | -0.04(-0.07– 0.00) 0.03 | -0.02(-0.05– 0.01) 0.15 | 0.002(-0.02– 0.02) 0.86 |
| Occasionally | -0.08(-0.11–-0.05) <0.001 | -0.06(-0.09–-0.03) <0.001 | -0.02(-0.04– 0.001) 0.48 |
| Frequently | -0.19(-0.21–-0.16) <0.001 | -0.12(-0.15–-0.10) <0.001 | -0.04(-0.06–-0.03) 0.002 |
| *P* for trend (character2integer) | <0.001 | <0.001 | <0.001 |
| Crude model: No adjustments at all | | | |
| Model 1: adjusted for age, sex, BMI, ratio of family income to poverty, and race or ethnicity | | | |
| Model 2: adjusted for age, sex, BMI, ratio of family income to poverty, race or ethnicity, smoking status, alcohol use status, Healthy Eating Index score, and physical activity  Abbreviations: HDL-C, high-density lipoprotein cholesterol; β: Coefficient; 95% CI, 95% confidence interval | | | |


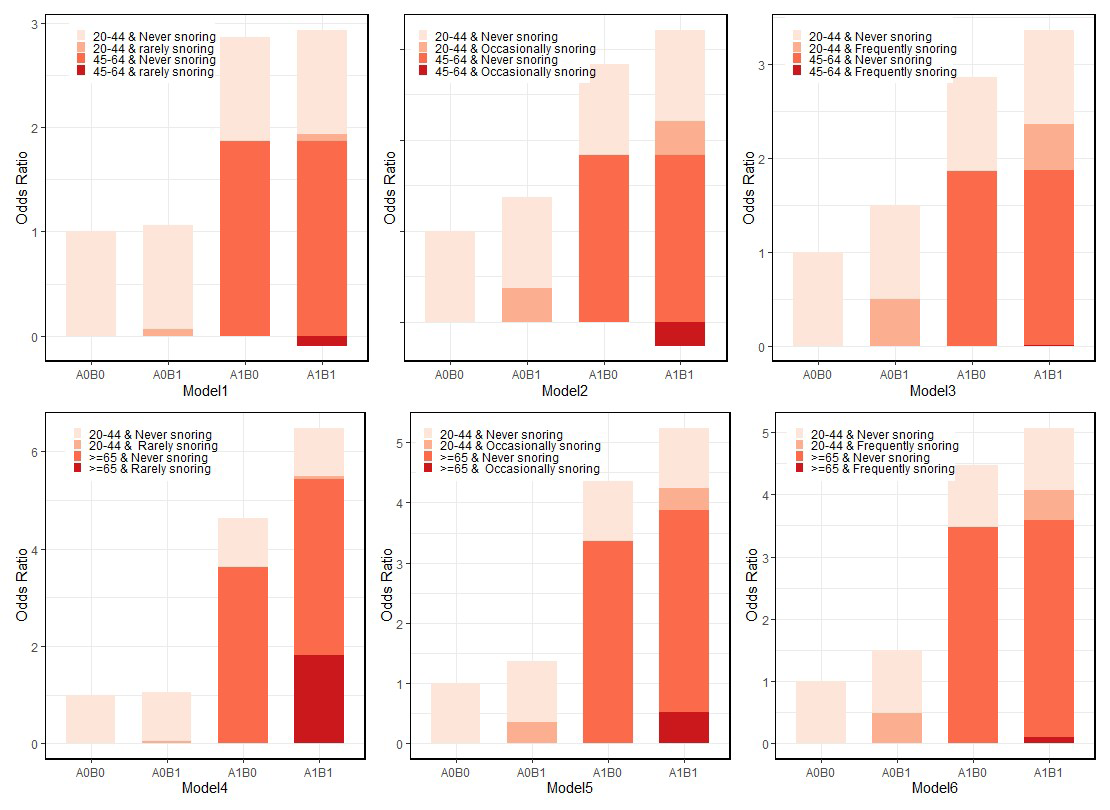


**Figure. S1**

Visualization of the interaction between age and snoring on dyslipidemia

The meaning of A0B0, A0B1, A1B0 and A1B1 were given in the corresponding legend. Model 4 was the only interaction item that maintained significance among the six models. Detailed analysis results of the six models were shown in Table S7.
